# Global Research Trends on Prostate Diseases and Erectile Dysfunction: A Bibliometric and Visualized Study

**DOI:** 10.3389/fonc.2020.627891

**Published:** 2021-02-12

**Authors:** Chengquan Ma, Hao Su, Hongjun Li

**Affiliations:** Department of Urology, Peking Union Medical College Hospital, Peking Union Medical College, Chinese Academy of Medical Sciences, Beijing, China

**Keywords:** erectile dysfunction, prostate cancer, benign prostatic hyperplasia, bibliometric analysis, radical prostatectomy

## Abstract

**Objectives:**

To identify the cooperation of authors, countries, institutions and explore the hot topics’ prospects regarding research of prostate diseases and erectile dysfunction (ED).

**Methods:**

Publications on research of prostate diseases and ED were retrieved from the Web of Science Core Collection (WoSCC). Bibliometric analyses were performed using VOSviewer and CiteSpace software. Network maps were generated to evaluate the collaborations between different authors, countries, institutions, and keywords.

**Results:**

A total of 2,599 articles related to study of prostate diseases and ED were identified. We observed gradually increasing in the number of publications from 1998 to 2016, and the trend was to be relatively stable in the past 3 years. Journal of Sexual Medicine (243 papers) owned the highest number of publications and Journal of Urology was the most co-cited journal. Mulhall John P (52 papers) was the top most productive authors and Mcvary Kebin T with the largest numbers of citations (1,589 co-citations) during the past decades. There were active collaborations among the top authors. The USA was the leading contributor in this field with 1,078 papers. Active cooperation between countries and between institutions was observed. The main hot topics included matters related to erectile dysfunction, prostate cancer, quality-of-life, radical prostatectomy, sexual function, and BPH.

**Conclusion:**

Bibliometric analysis provides a comprehensive overview of the development of scientific literature, allowing relevant authors and research teams to recognize the current research status in this field and at the same time provide a reference for formulating future research directions.

## Introduction

Prostate cancer (Pca) is one of the common malignant tumors of the male urogenital system. It is currently recognized as one of the most important medical problems faced by the male population. Its incidence ranks first of all malignant tumors in men (1). Radical resection of prostate cancer (RP) is currently the most important method of clinical treatment of localized prostate cancer, and it has a good long-term prognosis. Erectile dysfunction (ED) occurs in almost all patients after receiving RP and the incidence of ED after RP is 14~89% (2). A recent study followed up 326 patients with RP, the results showed that there is no significant difference in the scores of sexual function between robot-assisted laparoscopic prostatectomy and radical retropubic prostatectomy-surgery 6 months after surgery (3). It can be seen that ED is a common complication of patients after RP.

Benign prostatic hyperplasia (BPH) is one of the common diseases in middle-aged and elderly men. Studies have shown that BPH has an impact on the quality of life of about 50% of men over the age of 50 (4). Transurethral resection of the prostate (TURP) is still the gold standard of surgery. However, as a traumatic surgery, its impact on patients’ urinary and sexual functions has attracted more and more attention. Studies have pointed out that BPH and related lower urinary tract symptoms are closely related to the decline of erectile function. The more severe the degree of BPH, the more obvious the negative impact on erectile function (5). Therefore, the purpose of minimally invasive BPH surgery is not only to improve urination symptoms and it is more likely to improve the erectile function. The drugs used to treat BPH usually include 5a-reductase inhibitors to reduce prostate volume and improve LUTS symptoms. Data from a large number of clinical studies clearly showed that treatment with 5a-reductase inhibitors can reduce ED (6).

In prostate diseases, the majority of studies on the effects of ED were BPH and PCa. Usually, prostate diseases include Pca, BPH, prostatitis, and enlarged prostate may affect erectile function. A few studies have confirmed the effect of prostate volume (TPV) on ED, but the results of the studies are not consistent. A study from the British (7) included 427 volunteers found that there was no difference in the prostate volume measured by transrectal ultrasound between the no ED group, the mild ED group, and the severe ED group. In addition, several studies have shown that prostate volume enlargement is significantly associated with the risk of erectile dysfunction (8), and it has been found that prostate volume exceeding 30 cm^3^ is the best cut-off point for predicting ED (9). The study of Amel Kardasevic et al. (10) compared the IEFF scores according to the size of the prostate. The results showed that the volume of prostate is negatively correlated with IIEF-5 score. The increase of prostate volume leads to the decrease of IIEF score. Therefore, the prostate volume of patients with benign prostatic hyperplasia ED is very important, because the treatment of one disease may affect other disease, and *vice versa*. In addition, we also found that the history of prostatitis is also an independent risk factor for ED (11).

Bibliometric analysis refers to the use of mathematical and statistical methods to quantitatively analyze all the knowledge carriers of a certain discipline (12). Bibliometrics can qualitatively and quantitatively evaluate research trends based on the characteristics of literature databases and bibliometrics. It can not only help scholars grasp the development trend of specific research fields, but also evaluate the contributions of journals, institutions, and countries in specific research fields. And for the medical field, it can provide basis for the development of clinical guidelines (13, 14). This article aims to determine and study the characteristics of articles about prostate diseases and erectile dysfunction, summarize the current achievements in this field, grasp its research directions and hot spots, and provide certain references for future research directions (15).

## Methods

We performed online retrieval from the WoSCC database on Sep 22, 2020. We used the search queries to retrieve research articles between 1970 and 2020: TS = prostate AND TS = (impotence OR erectile dysfunction). We collected the following basic information for each article: title, abstract, authors, institution, country/region, journal, keywords, and references. Articles that meet the following criteria were included (1): The time span is between 1970 and 2020; (2) articles were indexed in WoSCC; (3) articles on research of prostate diseases and ED, including original research; The following documents were excluded: (1) irrelevant meeting abstracts, irrelevant case report, letters, irrelevant proceedings, corrected articles, and repeated articles; (2) systematic reviews, meta-analysis, and systematic review and meta-analysis; (3) unpublished documents without enough information for further analysis.

### Data Analysis

CiteSpace 5.7.R1 and VOSviewer 1.6.15 were used to perform co-occurrence analysis and visualize the collaborative networks of the authors/institutes/countries/keywords. Author co-citation analysis and reference co-citation analysis were also performed, and related knowledge maps were constructed. Burst keyword detection was also performed to investigate the recurrent new keywords. In this study, the 50 most cited or found articles were selected to create the individual network in a 1-year interval. Moreover, log-likelihood ratio weighting was used to analyze the contents of each cluster. A timeline view can depict changes in the trends of a field with time. In this study, the timeline was visualized using CiteSpace.

## Results

**Figure 1:**
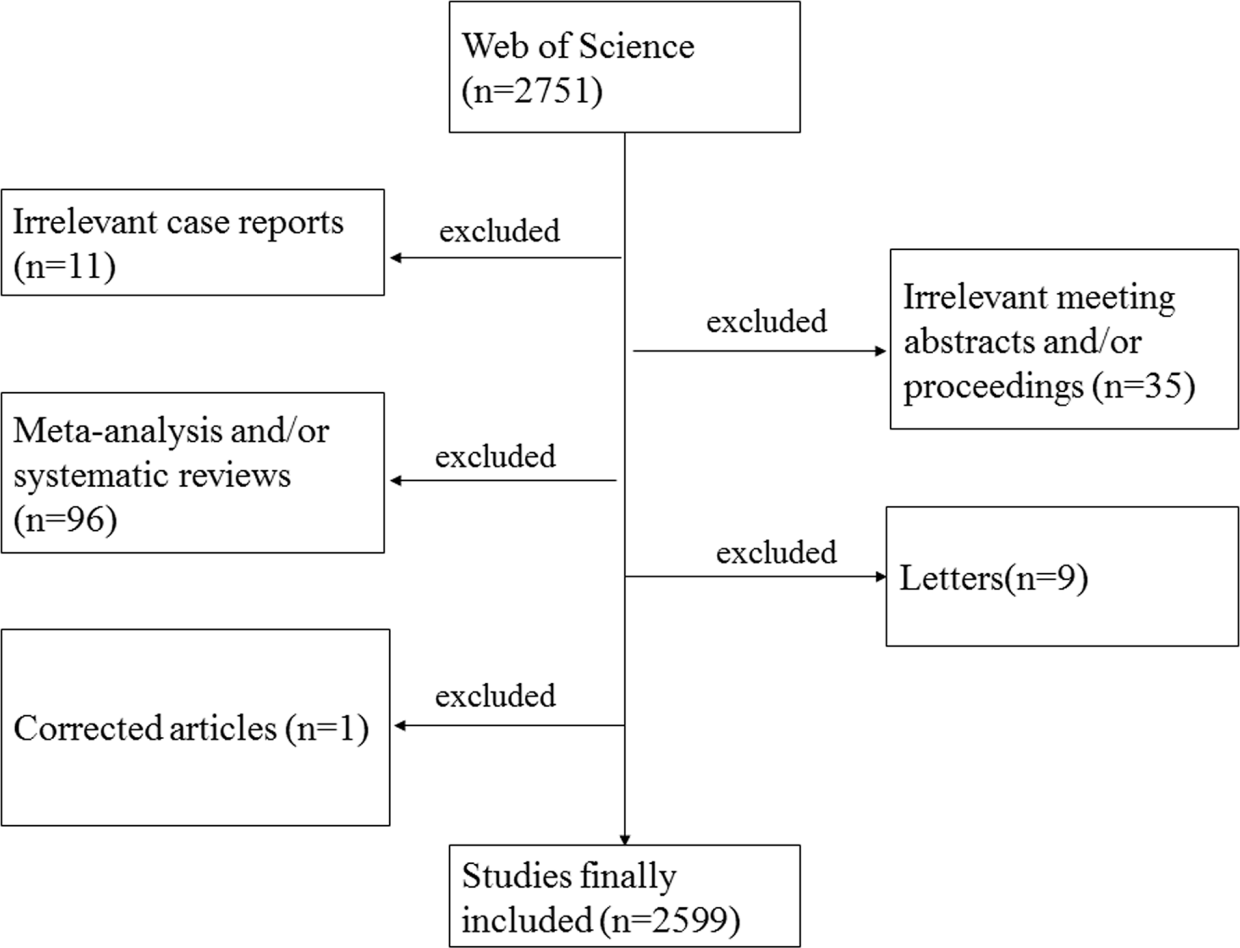
Flow diagram of the included papers.

**Figure 2:**
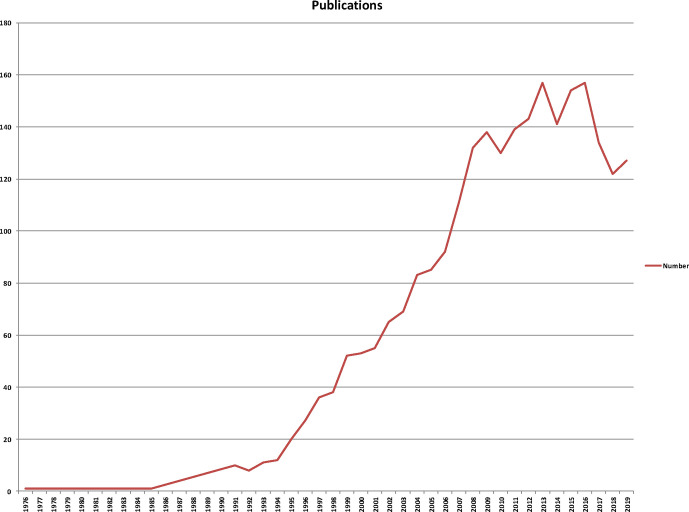
The number of publications by year and the overall trend consistently kept gradually increasing from 1998 to 2016, and the trend was to be relatively stable in the past 3 years.

In total, 2,599 papers matched the retrieval criteria (**Figure 1**). The number of publications by year was presented in **Figure 2**, where the overall trend consistently kept gradually increasing from 1998 to 2016, and the number of publications tended to be relatively stable in the past 3 years. Two thousand five hundred ninety-nine articles have been cited 84,495 times in total and the average number of citations per article is 32.51 times. In these published works, the sources of financial support include United States Department of Health Human Services, National Institutes of Health USA, National Institutes of Health USA, Eli Lilly, National Institute of Diabetes and Digestive and Kidney Diseases (NIDDK), UK Medical Research Council (MRC), Pfizer, American Cancer Society, National Natural Science Foundation of China (NSFC), National Institute for Health Research (NIHR), Astellas Pharmaceuticals, Prostate Cancer UK, etc.

### Analysis of Leading Journals and Cited Journals

In total, 446 academic journals have published papers about prostate diseases and ED research. **Table 1** presented the top 15 journals contributing to prostate diseases and ED research. Journal of Sexual Medicine as the leading journals published the most papers (243 papers), followed by Journal of Urology (227 papers), British Journal of Urology international (171 papers), Urology (163 papers), and European Urology (96).

**Table 1:** The top 15 productive and cited journals on the research of prostate diseases and ED.

Ranking	Journal	Frequency	IF (2019)	Cited (times)
1st	Journal of Sexual Medicine	243	3.293	6,391
2nd	Journal of Urology	227	5.925	15,110
3rd	British Journal of Urology International	171	4.806	6,260
4th	Urology	163	1.924	6,438
5th	European Urology	96	17.947	6,391
6th	International Journal of Impotence Research	82	1.388	1,953
7th	International Journal of Radiation Research	76	0.389	3,572
8th	World Journal of Urology	41	3.217	861
9th	Progres en Urologie	39	0.477	202
10th	Aging Male	38	0	825
10th	Cancer	37	5.772	2,272
12th	International Journal of Urology	33	2.445	555
13 th	Journal of Endourology	27	2.322	408
13th	Urologe	27	0.493	71
15th	International Urology and Nephrology	25	1.843	192

IF, Impact Factor.

**Table 1** presented the top 15 cited journals on prostate diseases and ED research. Journal of Urology was cited the most journals (15,110 times), followed by European Urology (6,710 times), Urology (6,438 times), and Journal of Sexual Medicine (6,391 times).

### Analysis of Leading Countries/Region and Institutions

**Figure 3:**
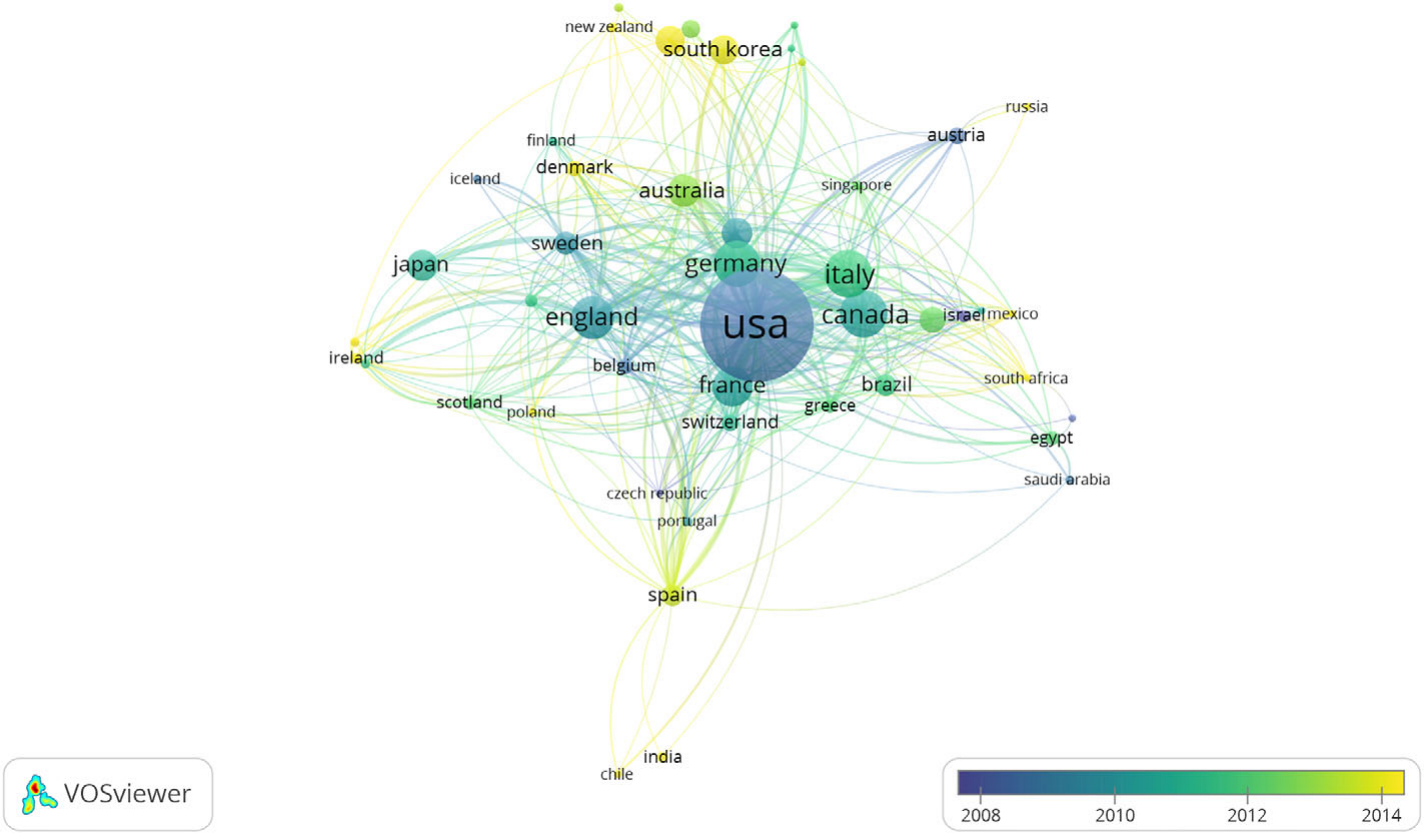
The font size of each country/region’s name represents the number of articles in the country/region. The thickness of the curved connecting line represents the collaborative intensity between countries/region; the country collaboration network of research on prostate diseases and ED; different colors inside the circle represent different time intervals.

**Figure 4:**
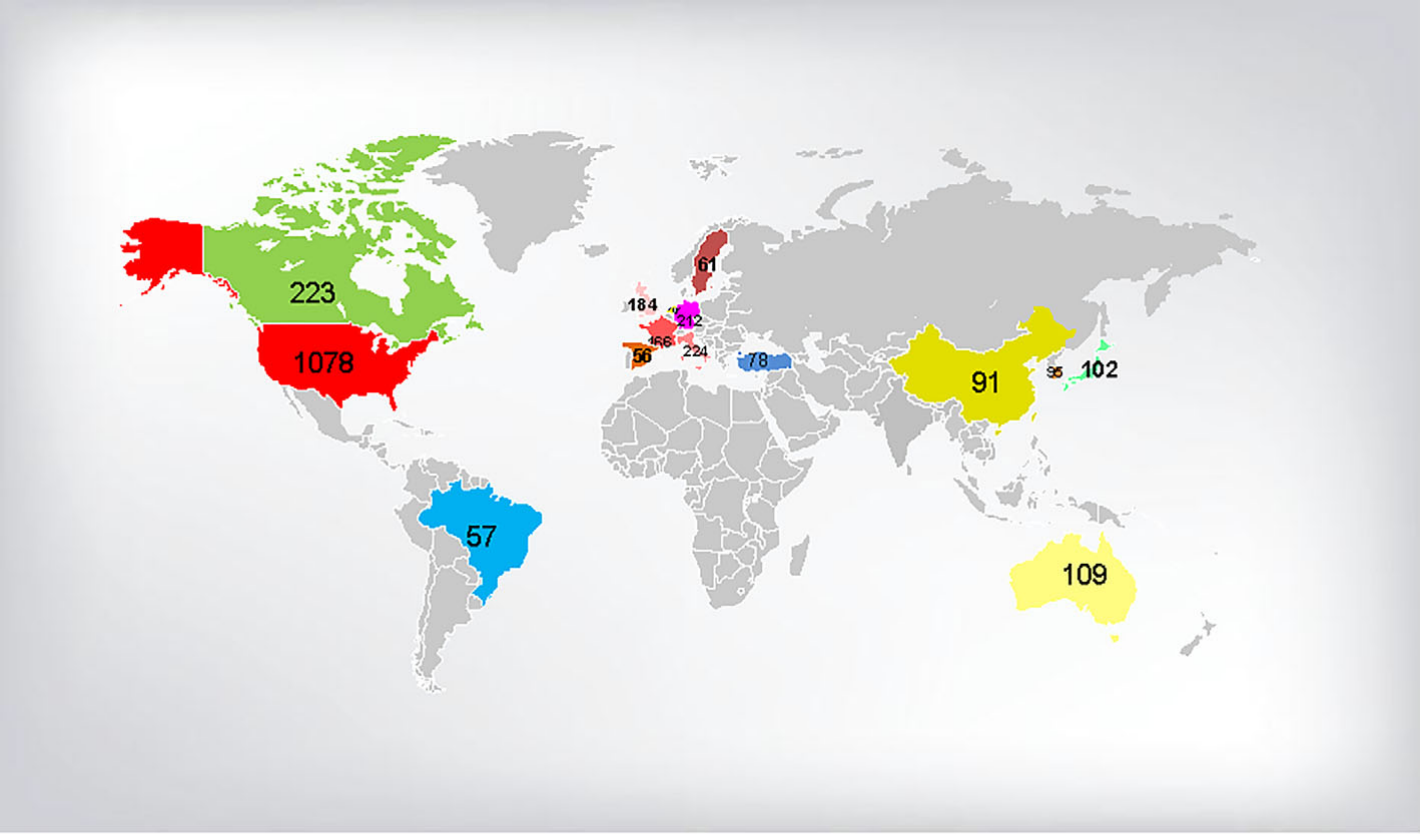
The global distribution of top 15 productive countries on the research of prostate diseases and ED.

The country/region collaboration network of prostate diseases and ED research was observed (**Figure 3**). **Table 2** and **Figure 4** presented the top 15 countries/regions contributing to prostate diseases and ED research. The USA contributed the most with the largest amount of publications (1,078 papers) related to prostate diseases and ED research, followed by Italy (224 papers), Canada (223 papers), Germany (212 papers), England (184 papers), France (166 papers), and Austria (109 papers). In the aspect of total link strength, the top five countries were USA (481), England (271), Germany (254), Canada (232), Italy (232), and France (169).

**Table 2:** The top 15 productive and cited country on the research of prostate diseases and ED.

Ranking	Country	Frequency	Cited (times)
1st	USA	1,078	49,975
2nd	Italy	224	8,329
3rd	Canada	223	6,988
4th	Germany	212	7,102
5th	England	184	9,004
6th	France	166	5,418
7th	Austria	109	3,821
8th	Jpan	102	1,812
8th	Netherlands	101	4,614
10th	South Korea	95	1,383
11th	People’s Republic of China	91	810
12th	Turkey	78	1,361
13th	Sweden	61	3,220
14th	Brazil	57	1,331
15th	Spain	56	1,461

**Figure 5:**
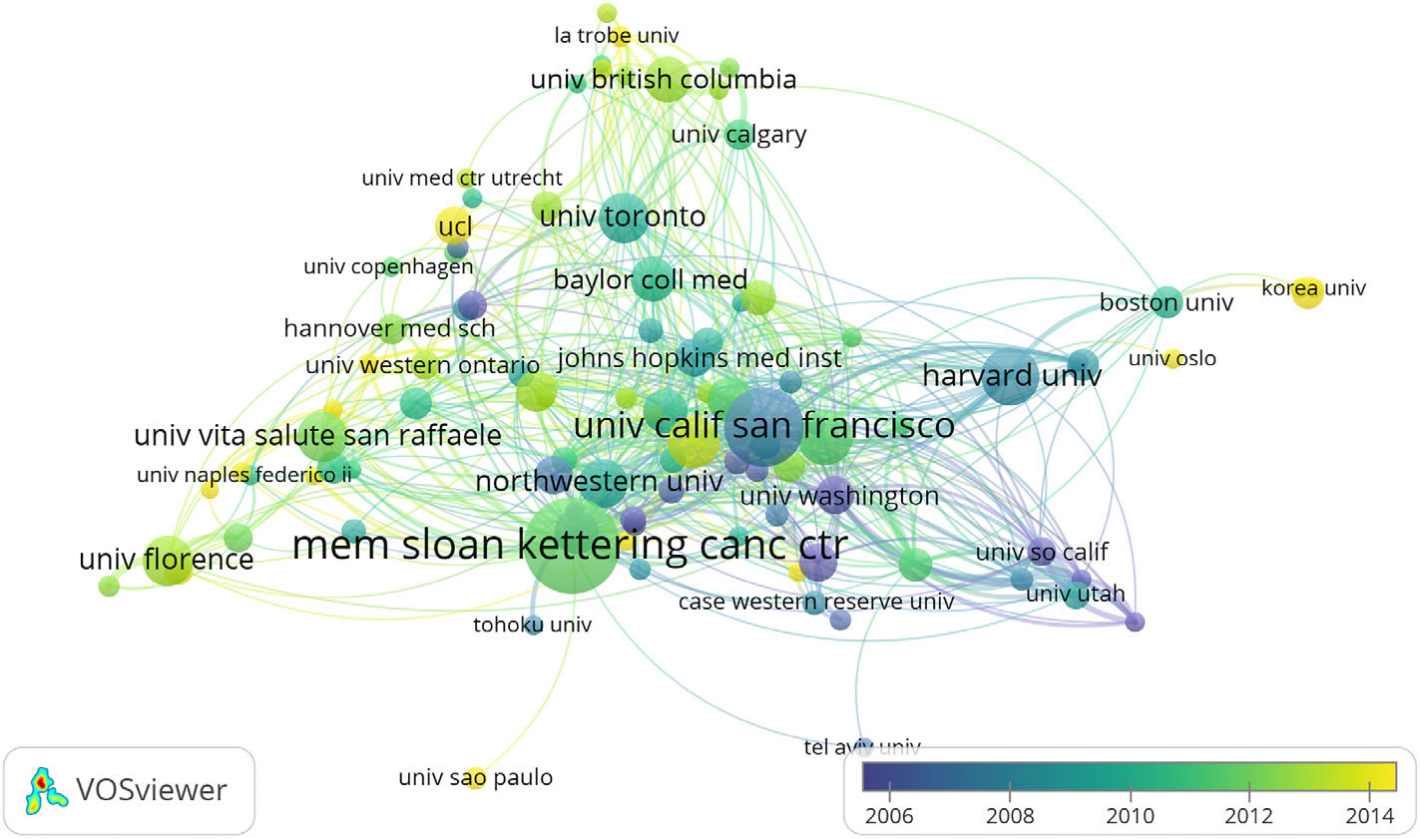
The font size of each institution’s name represents the number of articles in the institutions. The thickness of the curved connecting line represents the collaborative intensity between institutions; the institutions collaboration network of research on prostate diseases and ED; different colors inside the circle represent different time intervals.

The institution collaboration network of prostate diseases and ED was observed (**Figure 5**). The top five most productive institutions were Mem Sloan Kettering Canc Ctr (102 papers), Univ Calif San Francisco (78 papers), Harvard Univ (51 papers), University of Michigan (47 papers), and Duke University (43 papers), respectively.

### Analysis of Authors and Co-Cited Authors

**Figure 6:**
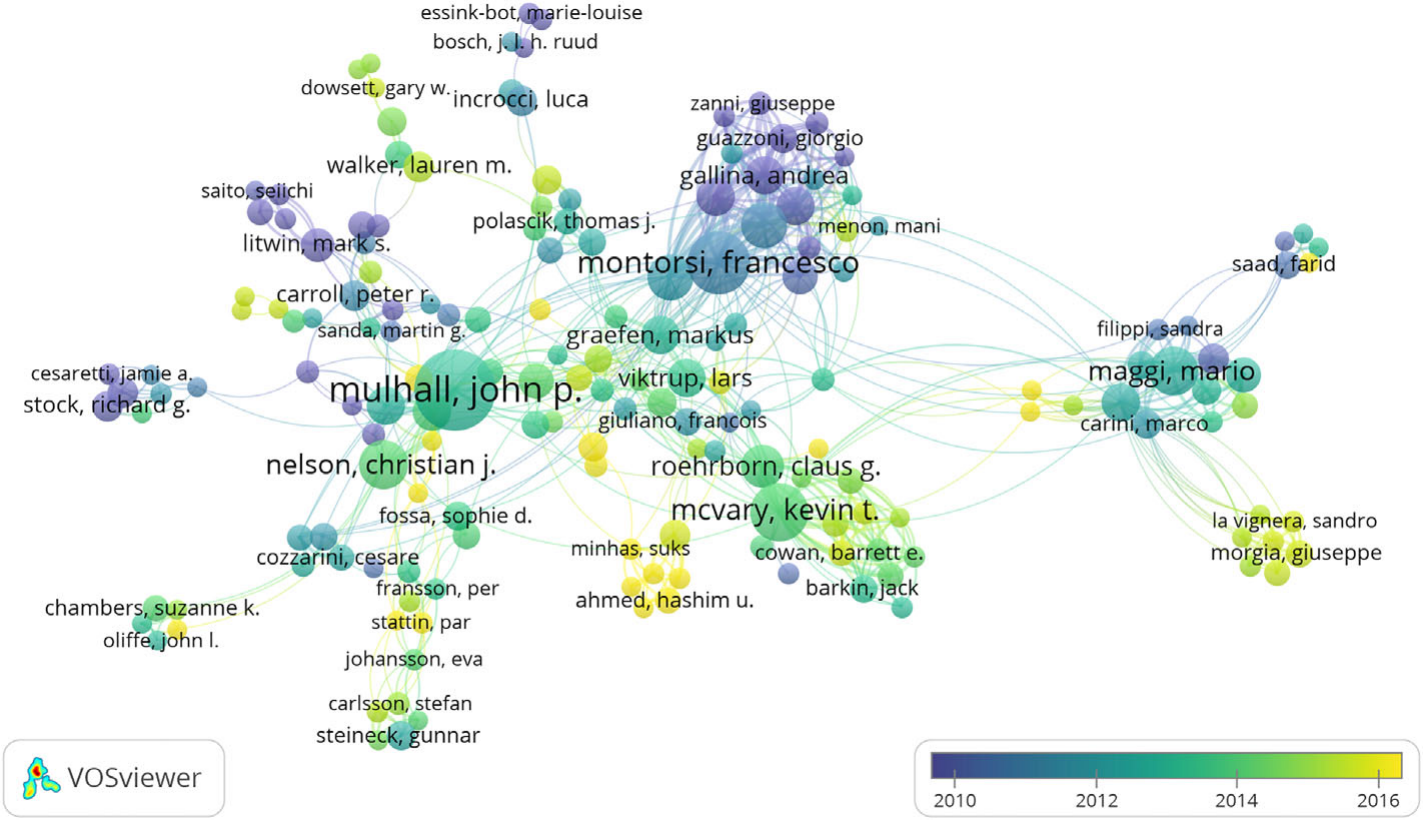
The font size of each author’s name represents the number of articles. The thickness of the curved connecting line represents the collaborative intensity between authors; the authors collaboration network of research on prostate diseases and ED; different colors inside the circle represent different time intervals.

The author collaboration network of prostate diseases and ED was observed. According to **Figure 6**, in terms of frequencies, Mulhall John P (52 papers), Montorsi Francesco (35 papers), Mcvary Kebin T (31 papers), Maggi Mario (23 papers), and Nelson Christian J (23 papers) were the top five most productive authors during the past decade. The information of author citation was also analyzed. In terms of co-cited author, the top five authors with the largest numbers of citations, which reflected their strongest academic authority, were Mcvary Kebin T (1,589 co-citations), Roehrborn Claus G (1,271 co-citations), Mulhall John P (1,258 co-citations), Emberton Mark (1,270 co-citations), and Maggi Mario (1,123 co-citations) (**Table 3**).

**Table 3:** The most productive authors on the research of prostate diseases and ED (Top 15).

Ranking	Author	Publications numbers	Cited (times)
1st	Mulhall John P	52	1,258
2nd	Montorsi Francesco	35	1,072
3rd	Mcvary Kebin T	31	1,589
4th	Maggi Mario	23	1,123
4th	Nelson Christian J	23	683
6th	Briganti Alberto	21	589
6th	Salonia Andrea	21	600
7th	Roehrborn Claus G	19	1,271
8th	Rigatti Patrizio	15	539
8th	Gallina Andrea	15	440
8th	Suardi Nazareno	15	448
8th	Gacci Mauro	15	510
8th	Graefen Markus	15	408
8th	Scardino Peter T	15	371
8th	Eastham James A	15	419
8th	Viktrup Lars	15	891

### Analysis of Co-Occurring Keywords and Burst Term

**Figure 7:**
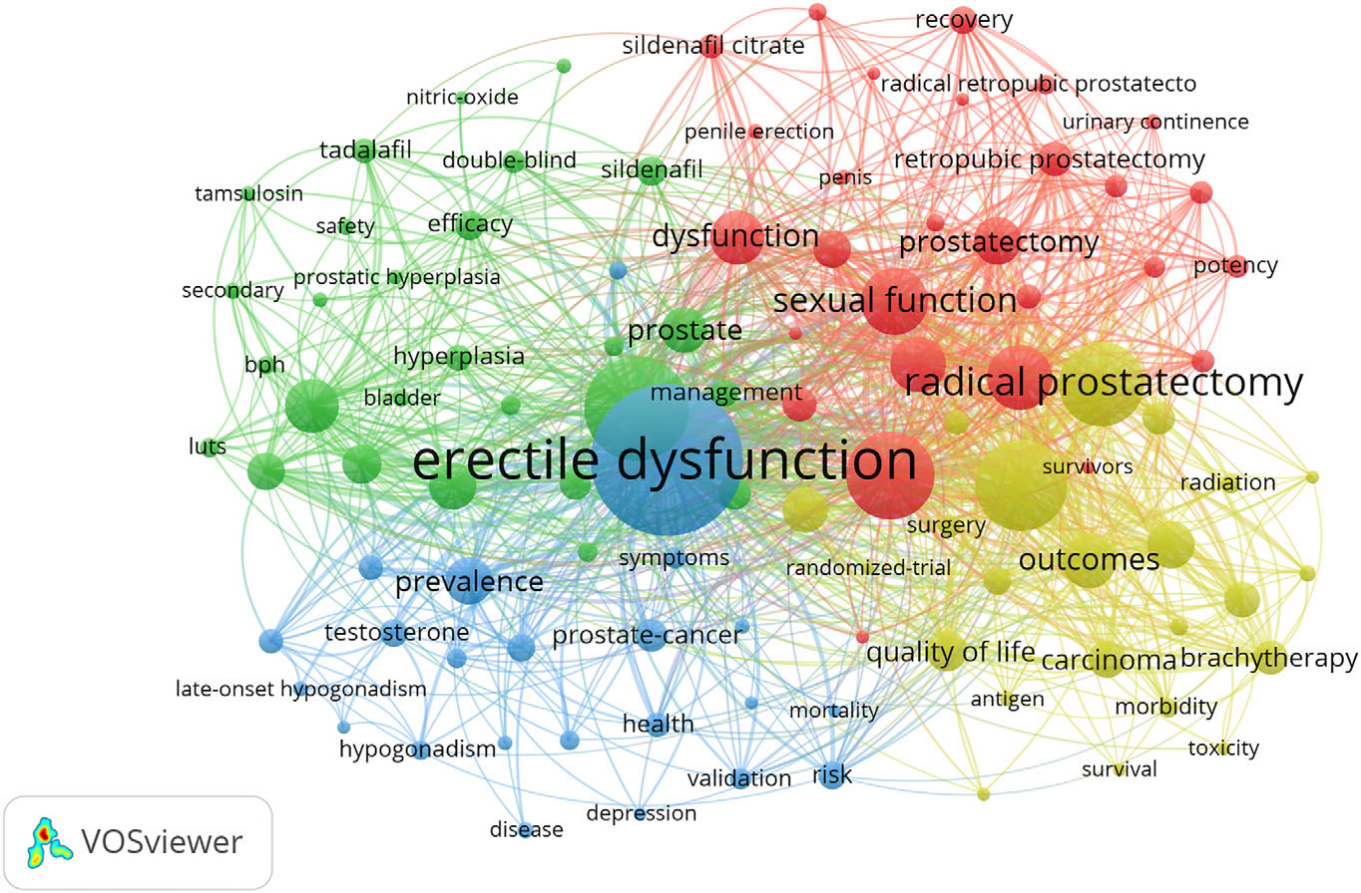
The font size of each keyword’s name represents the number of articles in the institutions. The thickness of the curved connecting line represents the collaborative intensity between keywords; the keywords collaboration network of research on prostate diseases and ED, and the same color represents the same cluster; the brighter the color, the more research about these keywords.

A total of 5,830 keywords were identified and the collaboration network of prostate diseases and ED as the show in **Figure 7**. The main hot topics about keywords included matters related to erectile dysfunction (1,263), men (755), prostate cancer (616), quality-of-life (596), radical prostatectomy (557), sexual function (402), cancer (387), impotence (311), dysfunction (304), and outcomes (301).

**Figure 8:**
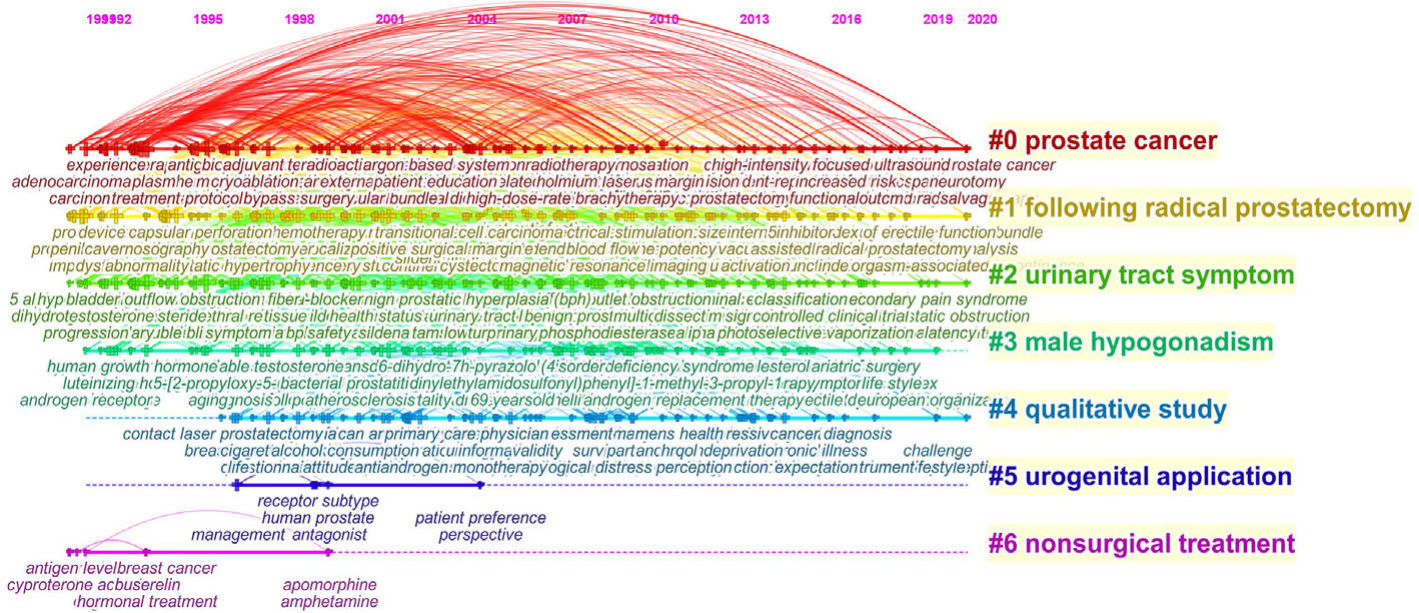
The timeline view of the knowledge map in prostate diseases and ED field. This view clearly presents the differences in the appearance time point and time span of seven clusters.

These words were classified into seven large clusters: “prostate cancer,” “following radical prostatectomy,” “urinary tract symptom,” “male hypogonadism,” “qualitative study,” “urogenital application,” and “nonsurgical treatment.” And the timeline view from 1990 to 2020 was shown as **Figure 8**, keyword time evolution of each cluster, from the initial research focus on “radical prostatectomy, erectile dysfunction” to the current research dimension of “pde5-I, surgical treatment” research changes.

### Co-Cited References and Burst References

We also revealed the top 12 co-cited references related to research on prostate diseases and ED. Three articles (Gormley 1992; Stanford 2000 and Rosen 2003) were co-cited more than 700 times, five articles (Sih 1997; Oelke 2013; Hu 2009, Thompson 2005 and Resnick 2013) were co-cited between 500 and 700 times, and 40 articles were also co-cited more than 200 times.

**Figure 9:**
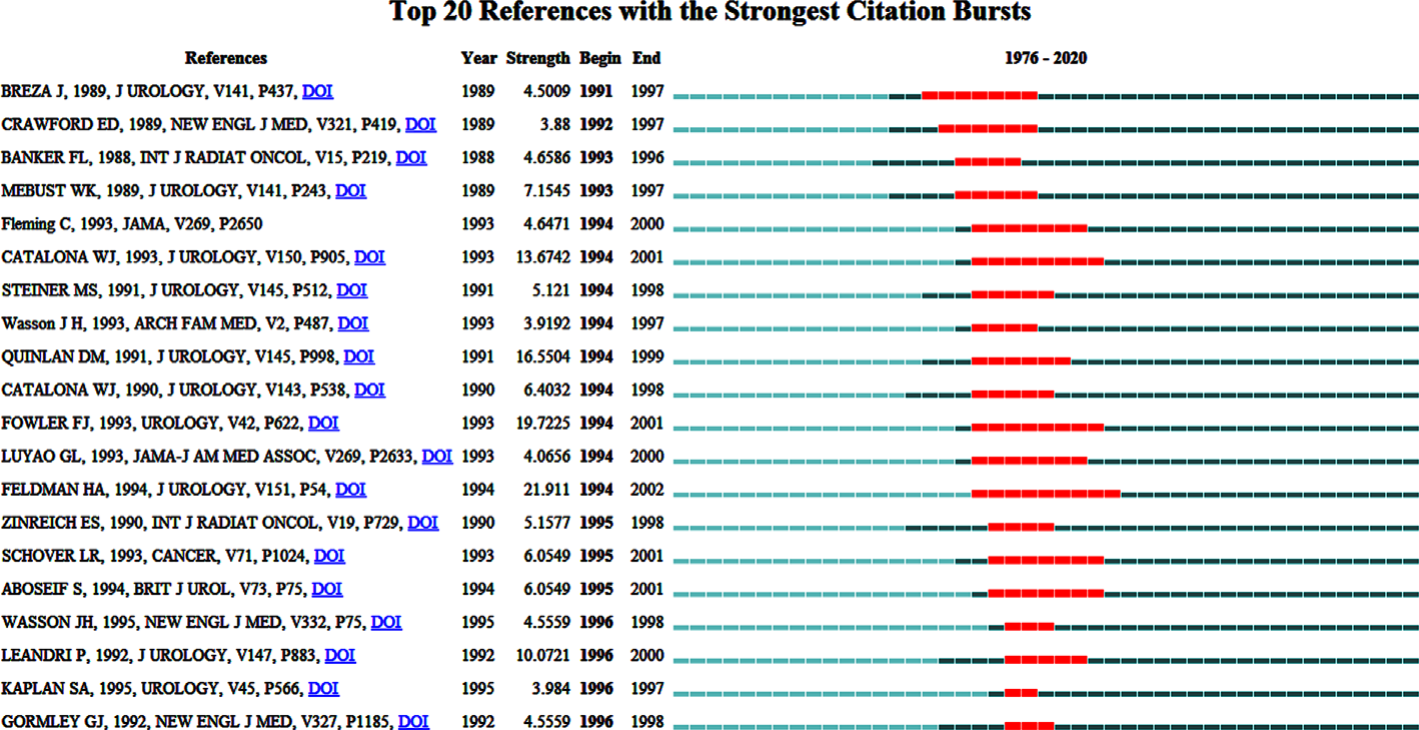
The top 20 references with the strongest citation bursts in the co-citation network.

**Figure 9** presented the top 20 references with the strongest citation bursts. Among them, the first reference with citation bursts appeared in 1989 (16); Most of the articles appeared citation between 1990 and 2002.

## Discussion

Prostate diseases and ED are common diseases in middle-aged and elderly men. Usually, ED is linked to aging, diabetes, cardiovascular disease, and some medications. The prevalence of ED in men over 40 years old is higher than 50% (17); however, prostate hyperplasia and tumors gradually increase with age, and ED often accompanies the emergence of prostate diseases. It is necessary to summarize the development of this subject. Therefore, relevant authors and research teams could recognize the current research status in this field; bibliometric analysis presents a comprehensive overview of the development of the scientific literature. This is the first application of bibliometric quantitatively and qualitatively methods regarding the prostate diseases and ED are involving 2,599 research papers retrieved from WoSCC.

The USA was the leading contributor in this field with 1,078 publications, followed by Italy, Canada, Germany, England, France, and Austria. The top five countries were all developed countries concentrated in Europe and America. The research output from these countries may be associated with the wide range of researchers with an interest within this field and a substantial amount of financial support to researchers. However, among the top 10 countries, only Japan and South Korea are from Asia, and the published literature is relatively less cited, although the population of Asia accounts for about 60% of the world’s total population (18). And we also found that the number of publications showed a gradually increased trend; this may be attributed to the growing economy of China and Chinese scholars’ access to more financial support (19, 20). In order to narrow the gap with developed countries in this field, we propose the following points: a. strengthen respect for technology and knowledge in the whole social environment; b. manager or executive branch of government to increase investment in the fund for scientific research; c. improve the assessment system for researchers, focusing on the number of articles and increasing the weight of authoritative journals.

From the perspective of the number of publications, the number of studies on prostate diseases and ED in journals showed a trend of increasing, and appeared to be relatively decreasing in the past 3 years. Literatures are funded mostly by the National Science Foundation of Country and major international pharmaceutical company, which indicate that research in this field pays more attention to in the national scientific and technological level. Related research published journals are relatively concentrated, Journal of Sexual Medicine published the most papers, followed by British Journal of Urology international, Urology and European Urology, etc. which are published in authoritative journals of andrology and urology. It can be observed that most of the papers published in this field are high-quality scientific research results. The average number of citations for each document is up to 32.51 times, which fully indicates that scholars can obtain international recognition in this field.

From the perspective of authors and institutions, Mulhall John P published the most papers and carried out the research on prostate diseases and ED earlier. Following Montorsi Francesco, Mcvary Kebin T, Maggi Mario, and Nelson Christian J were the top five most productive authors during the past decade, and they have cooperative relationships (21–23). However, it is found that the global research teams have obvious geographical features, and research teams were mainly from USA and Europe. Departments were concentrated in the andrology and urology of a university teaching hospital. Therefore, it is suggested to strengthen the communication and cooperation between the global cooperative research teams and look forward to more research results.

Keywords can provide immediate information about themes and the main theme in a particular study. Word analysis can show the concepts where a particular study focuses on. In our study, keywords co-occurrence analysis showed that the high-frequency keywords were mainly erectile dysfunction, men, prostate cancer, quality-of-life, radical prostatectomy, sexual function, cancer, impotence, dysfunction and outcomes, etc., indicating that the research focused on the impact on ED due to various types of prostate diseases (9, 11, 24–26). The majorities of these keywords were located in the middle of the network and were important as core words as they reflect the areas of interest in this field of research. Keyword cluster timeline map shows the keyword time evolution of each cluster, from the initial research focus on “radical prostatectomy, erectile dysfunction” to the current dimension of “pde5-I, surgical treatment” changes (27–30). This indicates that the research on prostate diseases and ED in recent years is being carried out at the level of treatments. And the most commonly used keywords in the last few decades can be preferred by researchers to access prostate diseases and erectile dysfunction studies in this field.

By systematically combining the literature in this field, this study shows the dynamic development process and structural relationship of relevant scientific knowledge through the atlas of scientific knowledge. It is suggested that the researchers in the field of prostate diseases and ED should follow the hot spot of scientific research, and the research institutions should strengthen the exchange and cooperation, so as to promote the academic development of this discipline. The analysis of this study is based on articles in WoSCC database. Although most of the research papers on prostate diseases and ED are included in this database, other databases such as Pubmed, Scopus may provide a broader coverage, which is a shortcoming of this paper. A majority of the included studies could be referred to follow-up update. As a consequence, they would cite previous studies on the same cohort and co-occurrence analyses could reveal collaboration between same authors (obviously) involved in serial analyses of the same dataset. At the same time, different analyses could derive from the same dataset. The latter could generate higher publication from a center and might have an effect on the analyses. In addition, the research results of this paper are completed based on software, and the machine algorithm is not as intelligent as the human brain in dealing with problems, which is prone to bias.

## Conclusion

The number of papers in this field had been rapidly growing since 1998 and remained relatively stable in the past 3 years. Papers published in a specialty journal will attract more attention than papers published in comprehensive journals in the field of prostate diseases and ED. The USA led in this field by contributing most of the total papers, and most of the top 10 countries were from the USA and Europe which greatly promoted the development of research. In order to narrow the gap with developed countries in this field, institutions and scholars in other countries/regions should innovate research methods to improve the quality of published papers. There was a close international cooperation between countries, institutions, and authors. It is normal to increase research results through international cooperation. Prostate diseases and ED therapy is a promising field of research that will benefit more patients. ED, prostate cancer, quality-of-life, radical prostatectomy, sexual function, and BPH are the current research hotspot and to use new therapy of prostate diseases or/and ED will reduce morbidity of ED and/or enhance efficacy of ED treatment.

## Data Availability Statement

The raw data supporting the conclusions of this article will be made available by the authors, without undue reservation.

## Author Contributions

CM and HL designed the study. All authors conducted the literature search and analyzed the data. CM wrote the paper. All authors contributed to the article and approved the submitted version.

## Conflict of Interest

The authors declare that the research was conducted in the absence of any commercial or financial relationships that could be construed as a potential conflict of interest.
